# Biochemical functional predictions for protein structures of unknown or uncertain function

**DOI:** 10.1016/j.csbj.2015.02.003

**Published:** 2015-02-18

**Authors:** Caitlyn L. Mills, Penny J. Beuning, Mary Jo Ondrechen

**Affiliations:** Department of Chemistry and Chemical Biology, Northeastern University, Boston, MA 02115, United States

**Keywords:** Structural genomics, Protein function prediction, Local structure methods, Computational chemistry

## Abstract

With the exponential growth in the determination of protein sequences and structures via genome sequencing and structural genomics efforts, there is a growing need for reliable computational methods to determine the biochemical function of these proteins. This paper reviews the efforts to address the challenge of annotating the function at the molecular level of uncharacterized proteins. While sequence- and three-dimensional-structure-based methods for protein function prediction have been reviewed previously, the recent trends in local structure-based methods have received less attention. These local structure-based methods are the primary focus of this review. Computational methods have been developed to predict the residues important for catalysis and the local spatial arrangements of these residues can be used to identify protein function. In addition, the combination of different types of methods can help obtain more information and better predictions of function for proteins of unknown function. Global initiatives, including the Enzyme Function Initiative (EFI), COMputational BRidges to EXperiments (COMBREX), and the Critical Assessment of Function Annotation (CAFA), are evaluating and testing the different approaches to predicting the function of proteins of unknown function. These initiatives and global collaborations will increase the capability and reliability of methods to predict biochemical function computationally and will add substantial value to the current volume of structural genomics data by reducing the number of absent or inaccurate functional annotations.

## 1 Introduction

The number of protein sequences and structures in databases such as UniProt [1] and the Protein Data Bank (PDB) [2] has grown significantly since the inception of genome sequencing and high-throughput structure determination. As of January 2015, the UniProt/TrEMBL database contains over 89 million protein sequence entries, an increase of more than six-fold since January of 2011; only a very small fraction of these proteins is assigned a reliable function [3]. Additionally, the PDB now includes more than 13,000 structural genomics (SG) protein structures as a result of structural genomics projects, notably the Protein Structure Initiative (PSI). At the turn of the millennium, the National Institute of General Medical Sciences (NIGMS) of the National Institutes of Health (NIH) in the United States launched the PSI with the goal to determine three-dimensional structures of proteins representing every family [4,5]. At that time, the human genome project and the sequencing of the genomes of many other organisms were completed [6,7]. The high throughput techniques developed by the PSI and other SG programs have increased the number of known protein structures. Since the PSI has been primarily concerned with high volume structure determination and prompt public availability of protein structures, most of these protein structures lack reliable accompanying information regarding their biochemical function; in some cases, no functional annotation is given. Thus, most of these proteins are assigned a putative or possible function based on the closest sequence or structure match; however, these assignments are often incorrect [8–10], and these incorrect functional labels can propagate within databases [11,12].

In 2010, the NIGMS launched a new phase of the PSI named PSI:Biology. This phase was implemented to determine the biological roles of the SG proteins under structural study. However, large numbers of functional annotations remain missing or incorrect. Better computational methods and verification through biochemical experimentation are clearly needed. Reliable and accurate computational methods for predicting the function of proteins can add significant value to genomics data and also improve efficiency of experimental verification of function. While there have been a number of review articles on sequence-based and three-dimensional-structure-based methods for function prediction [13–19], this article focuses on newer, local-structure-based computational methods to predict protein function at the molecular level; these methods are in turn based on prediction of the local spatial regions that are biochemically active in the structure. Finally, efforts within the broader scientific community to contribute to the testing and verification of functional predictions are explored.

When the function of a protein is not known, a putative function is sometimes assigned. These assignments are often the result of simple bioinformatics analyses including sequence and three-dimensional structure comparisons using programs such as BLAST [20,21] and Dali [22,23]. SG proteins can be assigned a putative function based on simple transfer of function from the closest sequence or structure match. However, sequence or structural similarities can be misleading. For instance, less than 30% of pairs of proteins with greater than 50% sequence identity have identical E.C. numbers [9]. Even a BLAST E value of 10^- 50^ or less does not guarantee that two proteins have the same function [9]. Sequence identities of 60% or greater will transfer function incorrectly in 10% of cases [10]. Furthermore, structural superfamilies, such as the enolase, amidohydrolase, and Clp/crotonase [24] superfamilies, can consist of several, or even dozens, of different biochemical functions [25–28]. The TIM barrel and the Rossmann fold each represent over 50 different types of biochemical function; the TIM barrel has been observed in five out of the six major E.C. categories and the Rossmann fold occurs in all six [29–32]. Thus, the practice of assigning function using simple transfer of function based on sequence or structure similarity has caused misannotations. In one study, the GenBank NR [33], UniProtKB/TrEMBL [1], and Kyoto Encyclopedia of Genes and Genomes (KEGG) [34] databases were shown to have up to 63% misannotation across six superfamilies [8].

For many SG proteins, possible functional assignments obtained from informatics-based approaches can provide too many options with insufficient discrimination of the most likely functions to be able to assign function with confidence or test function experimentally with reasonable efficiency. The development and implementation of new, reliable computational methods is an important aspect of a solution to the challenge of assignment of function to proteins.

## 2 Functional site prediction methods

Many computational programs have been developed to help predict the active sites and biochemical functions of proteins [16,18,19,35–39], although there remains much yet to be done to improve and to verify predictive capability for biochemical function.

### 2.1 Sequence-based methods

Sequence-based approaches are the more commonly used method of computational analysis [18]; these methods primarily utilize sequence alignments but sometimes also incorporate 3D structures [40,41]. Evolutionary Trace [42] and INformation-theoretic TREe traversal for Protein functional site IDentification (INTREPID) [43,44], examine a protein in its phylogenetic context and the evolutionary history of each amino acid in a protein sequence to assign a score to each amino acid. Evolutionary Trace analyzes the conservation of residues between proteins of similar function and evaluates amino acid variations that are known to be associated with changes in function. This information then suggests which residues are important for specific functions and which residues can be altered in order to change the function of a protein. This method exploits the similarities and differences between groups of homologous proteins and includes functional resolution, which involves analyzing the different functional clusters that are generated within a given family. Similar to Evolutionary Trace, INTREPID computes scores depending on the degree of conservation within a set of proteins with known functions. This score examines information over an entire family tree instead of just analyzing certain branches, or subfamilies. INTREPID is also able to identify residues important for catalysis that are not necessarily conserved across an entire family [44]. Both methods compute a score for each residue that is a measure of its importance to the function.

### 2.2 Structure-based methods

Structure-based methods of predicting protein function involve analyzing the structure and shape of a protein. This analysis helps determine where a ligand may bind by transferring the function of another similar protein of known function. Identification of the local site of biochemical activity in a protein can serve as a first step toward the prediction of the function. Geometric-based computational programs like Surfnet [45], CASTp [46], Ligsite [47], PocketFinder [48], and geometric potential [49] are structure-based approaches that examine the different properties of a protein surface or active site pocket to gain insight into the identity and location of binding pockets. Surfnet generates many protein surfaces, such as pockets within a protein, gaps between molecules, and van der Waals interactions, based on PDB coordinate data. The different surface output data are shown as a grid depicting the densities. These grids are created by applying a Gaussian function to the atoms within the protein. The residues that are specified as important are determined from the intensity of the densities. CASTp locates voids within a protein structure using the PDB, Swiss-Prot, and Online Mendelian Inheritance in Man (OMIM) to determine active site residues. Similarly, Ligsite uses a set of ligand–receptor complexes to locate pockets on a protein surface and can analyze a large set of proteins rather quickly [50]. PocketFinder and geometric potential also analyze the topological and geometric features of the protein surface. However, PocketFinder locates ligand binding envelopes instead of scanning the surface of a protein to find different sized pockets. Geometric potential adds local structural analysis in parallel with global structural analysis to analyze the residues within the pockets.

In addition to geometry-based methodologies, docking methods are another type of structure-based approach. Docking approaches such as Q-SiteFinder [51] and computational solvent mapping [52] identify the position and properties of the catalytic site regions within proteins through the use of small molecule probes. Q-SiteFinder exploits the energy differences between spaces in a protein and van der Waals probes. This helps find the locations in a protein that are energetically favorable for ligand binding. Similarly, solvent mapping uses small organic molecule probes to analyze a protein surface, locates favorable areas where the probes may bind, and then ranks the positions based on their free energies. These methods help locate both catalytic sites and non-catalytic, small molecule binding sites, such as allosteric sites within a given protein structure.

**Fig. 1:**
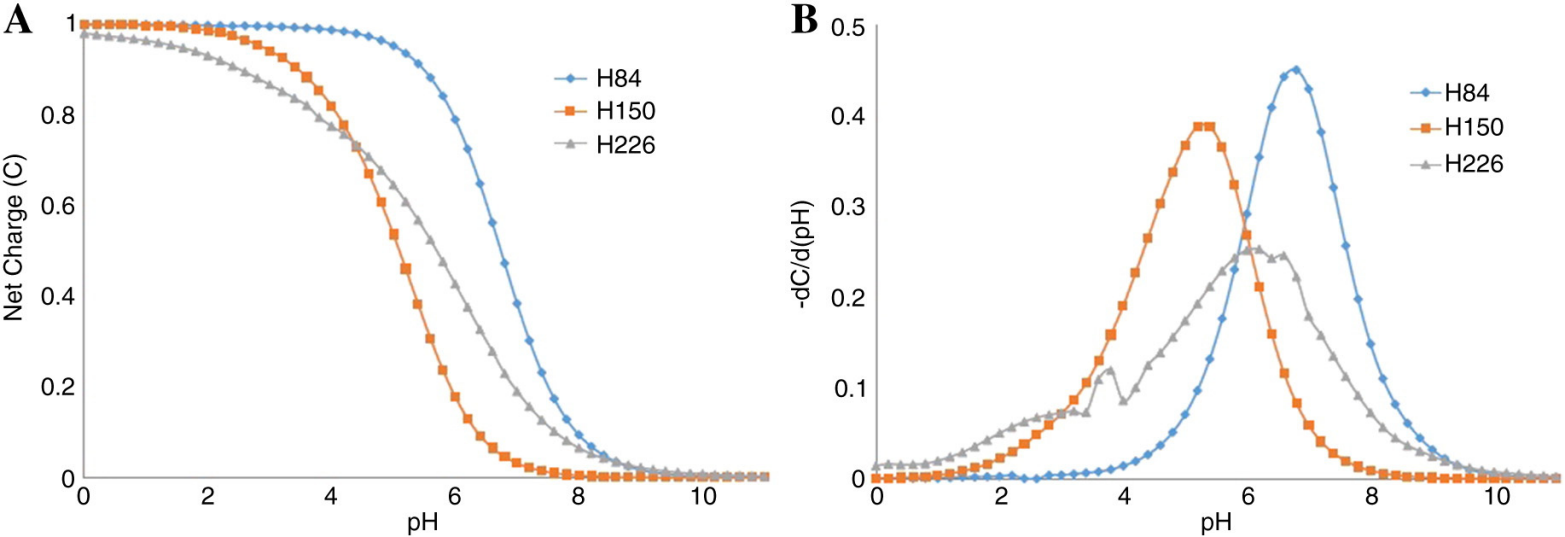
Three histidine residues from histidinol phosphate phosphatase (HPP) (PDB 2yz5) were analyzed by THEMATICS to produce theoretical titration curves (A), which plot the mean net charge of a given residue of a large ensemble of protein molecules as a function of pH, and first derivative plots (B).The titration curves of two non-catalytic residues, H84 and H150, show sigmoidal curve shapes with a small buffer range, while the catalytic H226 displays a curve with an anomalous shape, shallow slope, and larger buffer range. When analyzing the first derivatives of the titration curves, non-catalytic residues display symmetrical, highly peaked plots. However, active site residues such as H226 shown here display broad, asymmetric derivative plots and may have multiple peaks.

THEoretical Microscopic Anomalous TItration Curve Shapes (or THEMATICS) [53–55], a functional site prediction method, is able to predict accurately the ionizable active site residues within a given protein using only the 3D structure of the query protein. THEMATICS identifies ionizable amino acid residues (Arg, Asp, Cys, Glu, His, Lys, and Tyr, plus the N- and C- termini) that participate in catalysis or ligand recognition. The ionizable side chains of amino acid residues in protein active sites exhibit unusual electrostatic properties, specifically theoretical titration curves as shown in Fig. 1. These curves are obtained by approximate calculation of the electrostatic potential function, followed by a calculation of the average charge of each ionizable residue as a function of pH. These theoretical titration curves of active site residues are perturbed from the normal sigmoidal shape that is characteristic of the Brönsted acid–base chemistry of the free amino acid [53]. In a normal titration curve, the proton occupation is one at low pH and as the pH is increased, the proton occupation suddenly drops sharply around the p*K*_a_, approaching zero at higher pH. Normally this transition, where both the protonated and deprotonated forms exist in appreciable population, occurs in a narrow pH range. However, the residues within the active site tend to be partially protonated over a larger pH range and in this manner the shape of the titration curve is perturbed [53]. This method has been described previously as based on computed p*K*_a_ shifts [38,56]; however, this is incorrect. Only metrics that characterize the shape of the titration curves, and not the p*K*_a_ shifts, are used in the THEMATICS predictions. The degree of deviation of a catalytic ionizable residue from the typical Henderson–Hasselbalch titration curve can be quantified by the moments of the first derivative of the curve [57]. This method has been tested on the Catalytic Site Atlas (CSA) 100, and THEMATICS-predicted residues have been shown to constitute good predictions of the active site for proteins in the benchmark set [55]; they have also been shown to be generally well conserved [58].

### 2.3 Combined methods

In order to take advantage of the strengths of each approach to improve the performance of active site predictors, many current methods utilize structure and sequence-based properties in parallel [59–67]. ConCavity [68] is one method that utilizes both sequence and structure information to predict the functionally active residues of a protein. It uses algorithms that analyze not only the surface of a protein for binding pockets, but also uses evolutionary conservation to help locate these pockets. First, ConCavity scores areas on the surface of a protein according to the topology, using methods such as Ligsite or Surfnet (mentioned above). It then combines the conservation scores of residues within these pocket areas. Next, pocket structures are constructed based on the analysis and the structure of the protein. Finally, the potential pockets are mapped on the structure and the residues are analyzed and scored based on their position with respect to the pockets. With this information, ConCavity is able to predict spaces within a protein structure where a ligand is most likely to bind. The creators of ConCavity have shown that combining structure and sequence analyses significantly improves the ability to identify active site pockets and the residues responsible for catalysis [68].

Since THEMATICS can only predict the seven ionizable amino acids, machine learning methods were developed that can extract more information from the computed electrostatic and chemical properties and can predict all 20 amino acid types. The ionizable residues arginine, aspartate, cysteine, glutamate, histidine, lysine, and tyrosine make up about 76% of active site residues within functionally annotated proteins in databases [69]. To predict all 20 amino acid residue types, a new machine learning method was developed that can analyze the non-ionizable residues as well. This led to the development of Partial Order Optimum Likelihood, or POOL. POOL, a machine learning method, is a maximum likelihood, monotonicity-constrained multidimensional isotonic regression method that has the ability to identify both ionizable and non-ionizable active site residues [70]. POOL accepts THEMATICS metrics for the ionizable residues as one of its input features. However, it also calculates environment variables for all residues based on the THEMATICS metrics for the ionizable residues in the neighborhood of each residue. POOL can accept other input features, including scores from INTREPID [43,44] and the structure-only version of ConCavity [68]. Using structure-based geometric features, ConCavity supplies a score for each residue based on its likelihood of binding to a ligand. Together, these three input types from THEMATICS, INTREPID, and structure-only ConCavity generate POOL rankings that yield predictions of the residues that are important for catalysis.

For instance, THEMATICS and POOL were used to analyze the Structural Genomics protein *Bifidobacterium adolescentis* YP_910028.1 of unknown function and predicted that it is a metal-dependent phosphoesterase [71]. Sequence and structure comparisons with BLAST and Dali were inconclusive and suggested multiple different functions. The closest structure match was to a DNA polymerase catalytic domain. Initial phylogenetic analysis suggested that this protein could function to repair DNA or function as a DNA polymerase.

The crystal structure of YP_910028.1 contains a PHP domain, but PHP domains are present in multiple functional types, including X-family DNA polymerases [72], DNA polymerase III [73], and a histidinol phosphate phosphatase [74]. The location of the iron and zinc metals can suggest a general location for the active site, but cannot be used to determine a specific function since these trinuclear metal-binding sites are seen in a range of diverse proteins including endonucleases, phosphatases, and phospholipases [75–78]. Other analyses [79,80] were unable to provide a definitive functional annotation.

**Fig. 2:**
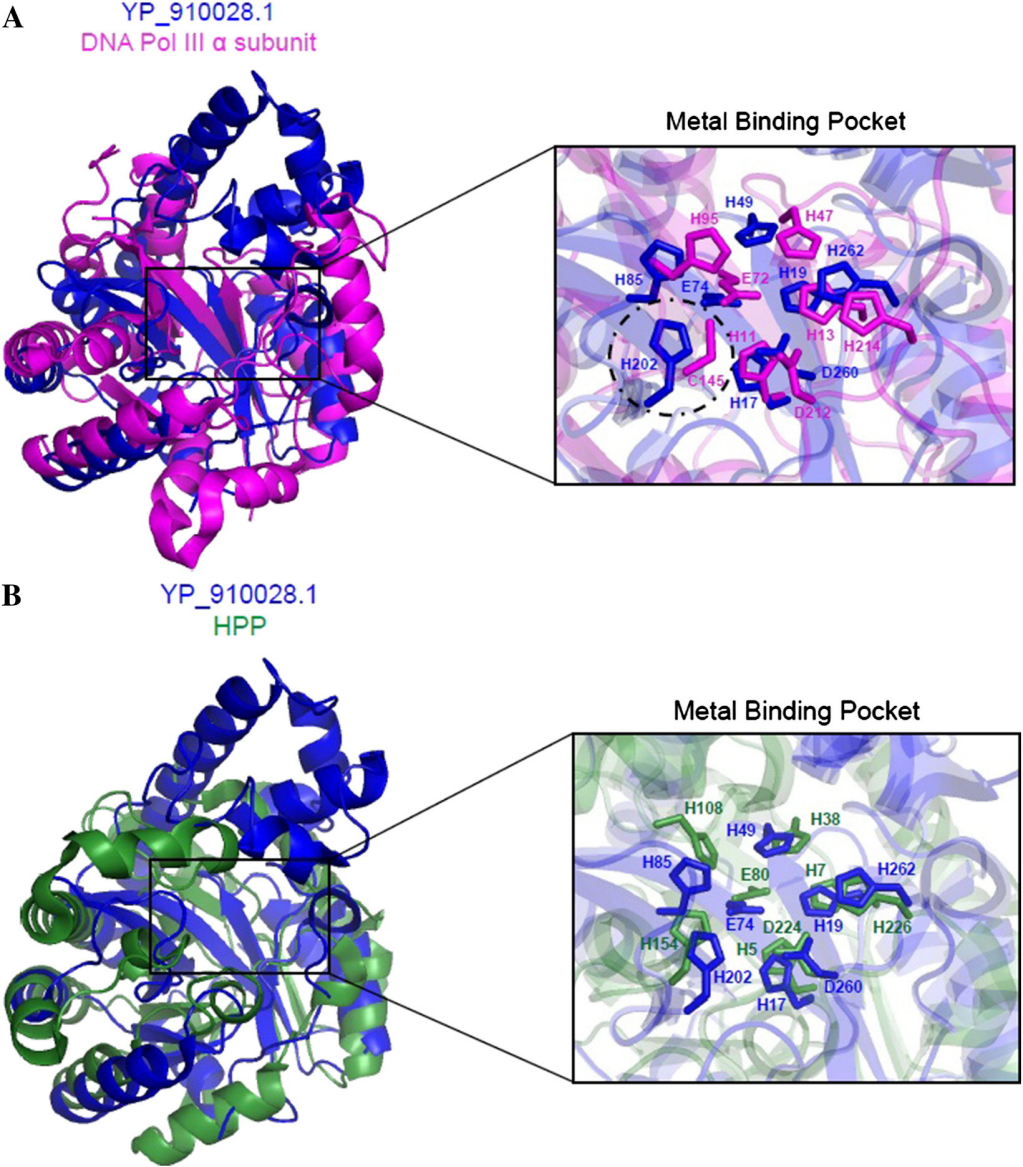
(A) The metal binding pocket of YP_910028.1, containing a PHP (Polymerase and Histidinol Phosphatase) domain (PDB ID 3e0f, shown in dark blue) aligns well with that of DNA Pol III alpha subunit (PDB ID 2hpi, shown in magenta). However, C145 and Y74 of DNA Pol III are mismatched with a histidine and threonine, respectively in YP_910028.1. (B) On the other hand, the metal binding pocket of YP_910028.1 (PDB 3e0f) aligns perfectly with the pocket of histidinol phosphate phosphatase (HPP) (PDB 2yz5), shown in green.

THEMATICS and POOL analysis of YP_910028.1 predicted sets of residues that closely match those predicted for histidinol phosphate phosphatase (HPP, PDB ID 2yz5) in a local structure alignment, with weaker matches to the other proteins of known function with similar folds, suggesting phosphoesterase activity for the enzyme. DNA polymerase III (PDB: 2hpi) has a similar metal-binding motif, but key cysteine and tyrosine residues are replaced by histidine and threonine residues in YP_910028.1, respectively. When YP_910028.1 is superimposed with both DNA polymerase III and HPP, the predicted active site residues align better with HPP (Fig. 2). This indicates that YP_910028.1 possesses phosphoesterase activity and not DNA polymerase activity. Phosphoesterase activity was detected by observation of the hydrolysis of the phosphate group of *para*-nitrophenyl phosphate (pNPP) to form *p*-nitrophenol and was shown to be dependent on the concentration of YP_910028.1. However, the tests for DNA polymerase activity resulted in no detectable activity regardless of the conditions used [71].

## 3 Annotating protein function

### 3.1 Local active site prediction methods

In comparison to global sequence- and structure-based methods that analyze an entire protein, local active site prediction methods find the biochemically active local region of the structure and then focus on the residues within the pocket and in the immediate surroundings. These methods are useful when analyzing entire families of proteins for which a specific signature is observed within the local active site.

For example, ProBiS [81] is a web server that utilizes an algorithm to detect similarities within protein binding pockets through local structural alignments of multiple proteins. ProBiS provides access to a database of 420 million pairwise local structure alignments and will perform pairwise local alignments for structures that are not in its database.

#### 3.1.1 ProFunc

**Fig. 3:**
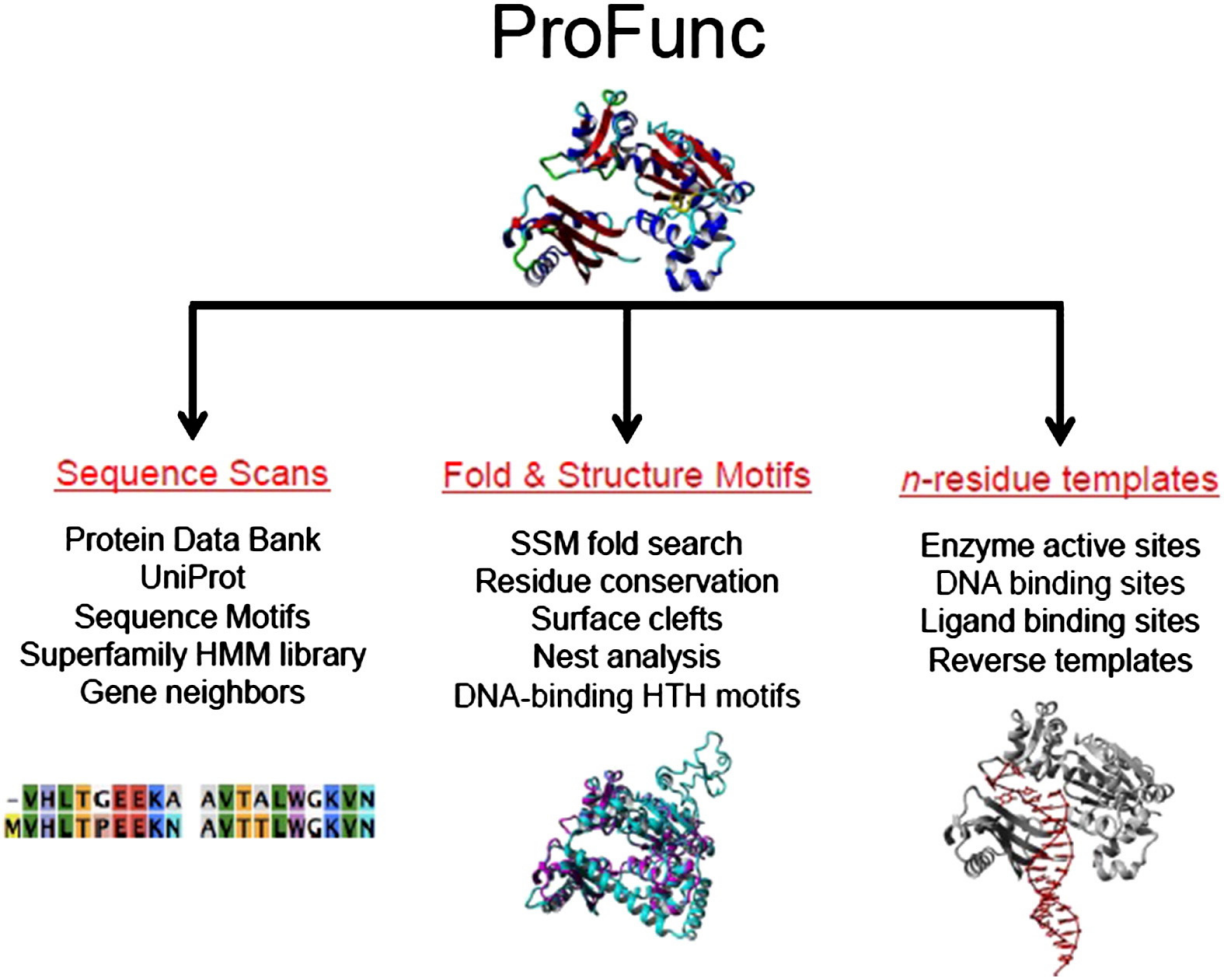
Schematic diagram outlining the different methods utilized in ProFunc. HMM: Hidden Markov Model; SSM: Secondary Structure Matching; HTH: Helix–Turn–Helix.

ProFunc [82] is a metaserver that combines sequence, global structure, and local structure-based methods to obtain a set of function predictions from which one might seek consensus. First, the protein of unknown function is analyzed by numerous sequence searches, shown on the left-hand side in Fig. 3. BLAST [20,21] analysis scans both the PDB and UniProt and uses multiple sequence alignment to determine sequence similarities and detect sequence motifs [83]. Gene neighbors are also examined based on the query protein's predicted location within the genome. The genes located near each other are often functionally related or functionally similar [82]. Next, structure-based analyses are performed on the protein of interest. This involves searching a number of databases for global folds or local structures that are similar to the query protein. Surfnet, mentioned in the above section, is one of these databases. Another database, secondary structure matching (SSM) [84] evaluates the secondary structure elements (SSEs) of the query protein of unknown function and compares them to the SSEs of protein structures within its database. The algorithm retrieves high, strong matches and superimposes the structures with the query protein to give a root mean square deviation (RMSD) so that a common number can be used to compare the results. Finally, ProFunc utilizes other servers to search for 3D templates of proteins with known binding sites. These binding sites may be simple active sites with the residues important for catalysis known [85], or ligand binding sites wherein residues important for catalysis are known and also the natural ligand/substrate is known. In some cases, the databases can also compare DNA-binding sites and motifs known to be associated with binding DNA.

#### 3.1.2 Structurally Aligned Local Sites of Activity (SALSA)

**Fig. 4:**
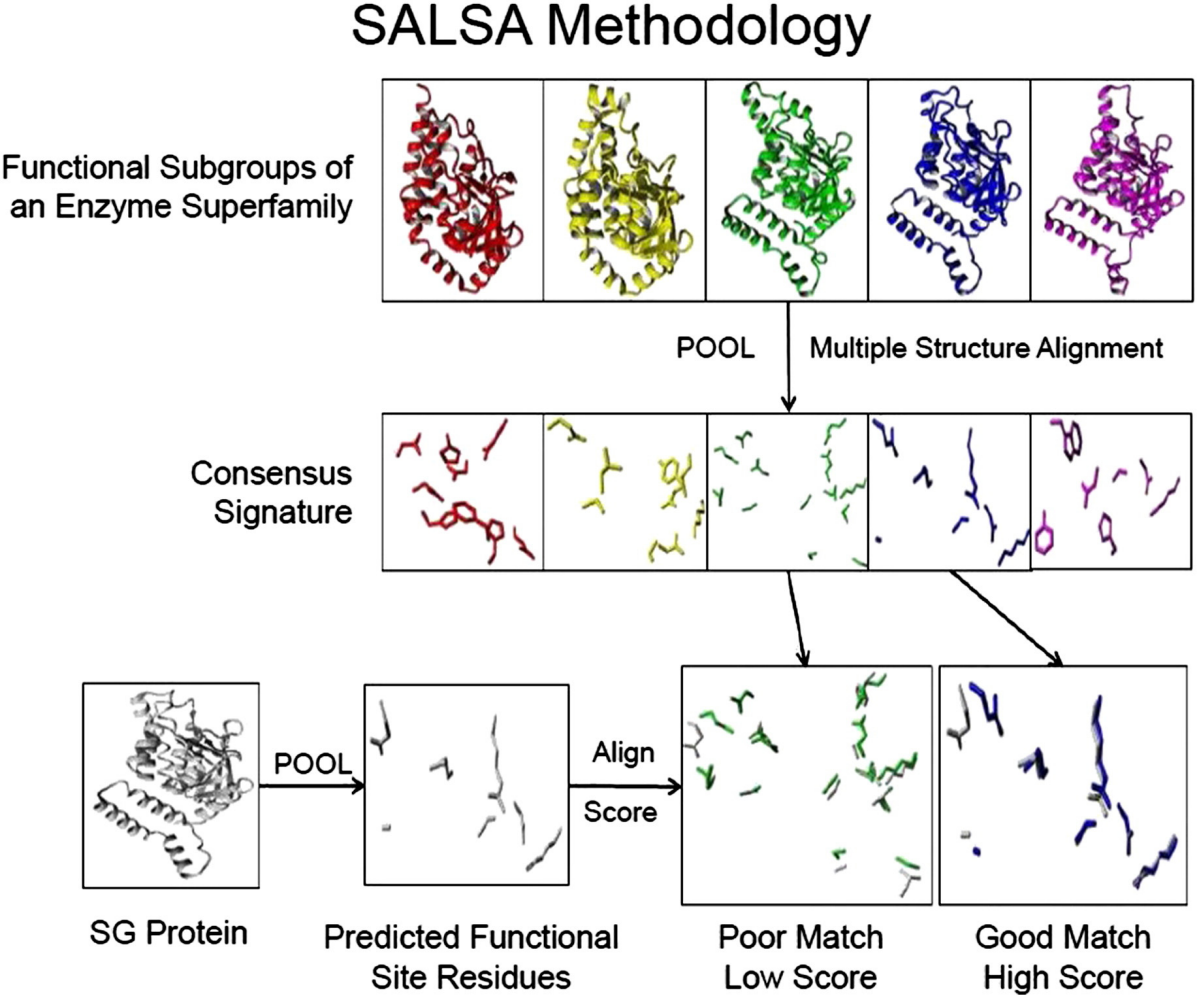
Schematic diagram outlining the SALSA method of annotating protein function.

The computational method Structurally Aligned Local Sites of Activity, or SALSA [86] utilizes a combination of functional residue prediction from POOL with local three-dimensional structural alignments. The characteristic spatial patterns of predicted residues at the local active site are used to identify biochemical functions. For example, a superfamily can consist of a number of functional families, each with a biochemical function that is different from the other members of that superfamily. SALSA tables can be constructed using the locally aligned residues at the predicted active sites across the entire superfamily. Proteins with the same function generally possess a particular spatial pattern or signature of predicted functional residues, while proteins of different functions possess different signatures. This consensus signature for each functional family is established using POOL predictions for a set of proteins with known common function; this defines the signature for each of the known functional types within a superfamily. If the superfamily contains SG proteins, the predicted sets of functional residues for the SG proteins can be compared with the consensus signatures for the known functional families. Thus, SALSA defines the different kinds of active sites, and therefore different functional types, within a superfamily. The general method is illustrated in the workflow shown in Fig. 4.

### 3.2 Community initiatives and projects

In an effort to tackle the growing challenges of protein function prediction and the correction of enzyme function misannotations within databases, the community has come together to take on the challenge. These global projects involve collaboration between numerous groups, employing theory, computation, and experiment, and have started to make significant progress toward the confirmation of protein function, thus adding a substantial value to the information on structural genomics proteins currently available.

#### 3.2.1 The Enzyme Function Initiative (EFI)

The Enzyme Function Initiative (EFI) [3], funded by NIGMS, began 10 years after the start of the PSI. This initiative combines bioinformatics with experimental enzymology to help determine the substrate specificity of proteins of unknown function. Each aspect of the EFI can be divided into whether or not the work can be done in a high throughput, moderate throughput, or low throughput manner. Generally, the first steps of the project, computational and bioinformatics analysis, fall under high throughput methods that help focus the experimental work in the final stages of this project, which involve lower throughput methods. The initial bioinformatics analyses, including database searches for sequences and structures of unknown function, preliminary molecular ligand docking, and clustering of pathways, can be executed on a high throughput basis [87]. Experimental enzymology, including preliminary homology modeling, expression and purification of enzymes of interest, and screening enzymes for different activities can be done at a rate of a few enzymes per month and falls under moderate throughput. The limiting factors of this project, however, are the experiments that fall under the low throughput category, including obtaining higher resolution homology models and docking studies, determining structure–function relationships, in vivo studies of functional predictions, and identification of enzymes with functional promiscuity [88,89], each of which can be highly demanding of time and labor. However, the preliminary work helps refine the experimental analysis, which highlights the necessity of reliable computational prediction methods to be used in parallel with experimental validation methods.

The project focuses these methods on five superfamilies with diverse functions that have been selected as test cases for developing the strategy outlined above: (1) amidohydrolase (AH), (2) enolase (EN), (3) glutathione transferase (GST), (4) haloalkanoic acid dehalogenase (HAD), and (5) isoprenoid synthase (IS). These Bridging Projects help determine target enzymes as well as information about the enzymes of known function in each superfamily.

In order to be successful, the EFI strategy must be able to assign a novel function for enzymes that are functionally diverse from enzymes of known function. However, molecular docking of a ligand into an enzyme is not always a reliable way to determine substrate specificities. In particular, substrates can cause conformational changes in vitro that are not observed in silico and the scoring algorithms may not be accurate [3]. At the end of its term, the EFI proposes that it will have a working strategy consisting of a set of databases and programs that the scientific community can utilize in expanding this analysis to every protein superfamily.

This method has been successfully tested on numerous proteins of unknown function. Specifically, the in silico docking method of the EFI described above has been successfully applied to the entire dipeptide epimerase family within the EN superfamily. Within this superfamily, a member of the *cis,cis*-muconate lactonizing enzyme (MLE) family encoded by the *Bacillus cereus* ATCC 14579 genome with previously unknown function was predicted to have *N*-succinyl arginine racemase function based on docking approaches [90]. A virtual library consisting of *N*-succinyl amino acids and dipeptides was virtually docked into a homology model of this enzyme. The homology model was created using a series of template structures from the PDB. The structure of l-Ala-d/l-Glu epimerase from *Bacillus subtilis* (PDB ID 1TKK) was the template that contributed the most to the homology model. This template was also prominent in many subsequent homology models for members of the dipeptide epimerase family and was useful in the docking studies of nearly 700 enzymes.

**Fig. 5:**
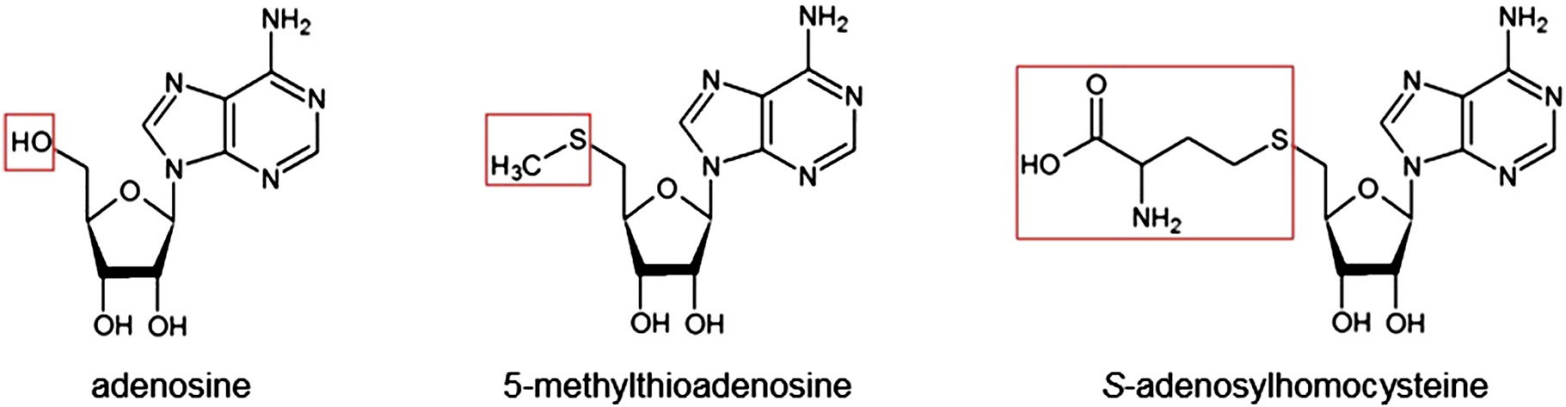
The metabolites above dock in silico into Tm0936 and are substrates of the enzyme Tm0936. The general structure of these three metabolites is the same with the exception of the moieties shown in the boxes.

Another successful docking study, performed by one of the Bridging Projects, aided in assigning function to *Thermotoga maritima* Tm0936, a member of the AH superfamily whose function was previously unknown. Tm0936 was predicted to have a novel function as an *S*-adenosylhomocysteine deaminase [91]. This study involved docking thousands of metabolites into Tm0936 and creating a target list comprising adenine analogues. Five potential substrates were chosen based on availability and rank within the docking study; of these, the enzyme had significant activity with three: adenosine, 5-methylthioadenosine (MTA), and *S*-adenosylhomocysteine (SAH) (Fig. 5). It was concluded that this enzyme is involved in the deamination of metabolites within the MTA/SAH pathway.

#### 3.2.2 Critical Assessment of Function Annotation (CAFA) experiment

Until recently, there was no way to compare the performance of different automated function prediction methods. Over the past few years, Iddo Friedberg and Predrag Radivojac, through collaboration with many computational research groups, have designed an experiment to test multiple automated function prediction tools and programs. This Critical Assessment of Function Annotation (CAFA) [92] experiment is a large-scale community-wide collaboration designed to evaluate the performance of the many diverse methodologies [60,82,93–99] developed by research groups over the years. These methods range from studying protein–protein interactions [100–103] to analyzing sequences [104–108] to examining evolutionary features of proteins [109–113]. The main focus is to evaluate the quality of current sequence-based automated function prediction methods and to identify the computational methods that perform the best in predicting correct or novel functions.

So far, the CAFA experiment has gone through two experimental periods, with the second experiment recently completed. In both instances, the protocols, or “rules,” are similar. The classification system used by the CAFA experiments was developed based on the definition of protein function classification by the Gene Ontology (GO) Consortium [114]. The GO project utilizes many different databases [115–142] to help provide a solution to the problem of automated function prediction. The main goal of the GO Consortium is to develop a uniform vocabulary to use when describing the functions of all eukaryotic proteins. The first CAFA project lasted 15 months and consisted of 30 teams of researchers from around the globe, who tested over 50 algorithms designed to annotate protein function. The different methods were tested on a set of over 860 protein sequences spanning 11 species, including *Escherichia coli*, *B. subtilis*, and *Homo sapiens* [92].

From the GO Consortium categories, this project involves information from the “Biological Process” and “Molecular Function” sections. These sections are two of the three structured vocabularies that the GO project has developed to describe gene products. The experiment began with providing a set of over 48,000 proteins of uncertain biochemical function to the teams involved. After the teams worked on annotating these proteins, the assessors performed GO experimental annotations over the course of almost a year. Of the protein sequences analyzed, a set of 866 were chosen based on the accumulation of functional annotations made over the 11 month period. The published results revolve around a maximum *F*-measure, also known as *F*_max_, which corresponds to a “harmonic mean between precision and recall” [92]. Two methods, BLAST [20,21] and a Naïve baseline method [92], were used to compare the test methods. In the BLAST method, the GO terms that define any protein sequences for which a function has been experimentally determined are assigned to the sequence being analyzed. In the Naïve method, the GO terms used to describe the target sequences are scored based on how frequently the term comes up in the Swiss-Prot database overall.

This large-scale CAFA experiment and others to follow like CAFA2 are designed to help researchers evaluate their methods in comparison to other methods in existence. They also provide the community with a set of predictions for a number of proteins of unknown or uncertain function. Overall, the results of the first experiment showed accurate performances when predicting the “Molecular Function” of the target proteins. However, the same could not be said for predicting the “Biological Processes” of the target proteins, which shows room for improvement in all methods.

The two top performing methods for predicting both “Molecular Function” and “Biological Process” ontologies were Jones—UCL [143] and Argot2 [144]. The Jones—UCL method uses known protein–protein interactions, gene expression, and sequence similarity to assign protein functions [143]. The Argot2 method analyzes a given protein sequence by BLAST [20,21] and HMMer [145,146] first, followed by a search of GO terms from the UniProtKB-GOA database [138]. The results highlight the improvement in function prediction that can be gained from combining multiple input features.

In the first CAFA global project (CAFA1), an analysis of human mitochondrial polynucleotide phosphorylase 1 (hPNPase) from a family of exoribonucleases was reported. This large protein works in complex with other portions of the mitochondrial degradosome and is characterized by a number of diverse functions for which experimental data exist. These functions include hydrolyzing single-stranded RNA [147], facilitating the import of RNAs into the mitochondrial matrix [148], and responding to oxidative stress [149]. A number of methods under examination in the CAFA project made predictions for hPNPase. In the “Molecular Function” GO terms category, most methods were able to predict correctly two functions for hPNPase: single-stranded RNA hydrolysis and import of small RNAs. Other functions are more uncommon within the family of hPNPase, which may contribute to the lack of methods able to predict these functions [92]. The most well-known biological function of hPNPase is the import of RNA into the mitochondria. Within the “Biological Process” GO terms category, this major function as well as others were not predicted.

#### 3.2.3 COMputational BRidges to EXperiments (COMBREX)

The COMBREX Project's main goal is to understand and annotate the function of microbial proteins [150]. As its name implies, this project brings theorists and experimentalists together in order to increase the rate at which proteins from archaeal and bacterial genomes are functionally annotated [151]. There are three main components to this project: the *COMBREX Community*, the *COMBREX Database*, and the *COMBREX grants*. The grants are used to fund community members working on the efforts described above, while the database serves as a universal place to house the list of functionally annotated proteins. Currently, this database contains more than 3.3 million proteins spanning over 1000 microbial genomes [150]. Of the genes in the database, less than 0.5% have experimental data regarding the function of the gene. However, over 75% of the genes contain a computationally predicted function, but lack experimental validation. In general, the COMBREX project is working toward creating a Gold Standard Database to serve as the basis for training algorithms for future protein annotation methods. During the beginning of the COMBREX-funded projects, experimentalists were assigned 140 proteins on which to perform experiments. Of these 140 proteins, 37 contain 28 unique domains that are similar to human proteins, which potentially can lead to new information about human health and diseases. Also within this group of proteins are eight domains of unknown function defined by Pfam, which allows for some novel predictions of function to be made. Of these 140 proteins, about half have a successfully validated functional prediction [152–156]. In one instance [155], bacterial YbbB is identified in twelve archaeal genomes and its function is determined to be a tRNA 2-selenouridine synthase. In order to confirm this functional classification, first preliminary computational analysis, including BLAST [20,21] searches, was performed on the protein of uncertain function. Next, structure-based alignments and neighboring genes were analyzed using CLUSTAL W [157] and a neighbor-joining method [158]. To validate the results of the computational methods, in vitro activity assays were performed by gene complementation/replacement [159,160] and tRNA selenation [155] experiments. In the end, the computational predictions were successfully validated by the experimental methods, and the function of this protein was determined.

## 4 Summary and outlook

The process of annotating proteins of unknown and uncertain functions continues to be challenging yet critical for understanding the enormous amount of information generated by genome sequencing and structural genomics projects. Function prediction methods that focus on the local spatial region of biochemical activity show promise for improving predictive capability. Proteins that contain high sequence similarity on a global level do not always have that same sequence similarity at the local active site. Conversely, proteins with low overall sequence similarity can have high similarity in the spatial region of the active site. Too often, the function of a protein that has high global sequence similarity with a protein of unknown function is transferred to the target protein without analyzing the local active site sequence similarities.

In an effort to provide useful information about enzymes of unknown function, many research groups have developed methods to predict protein function. However, the probability of misannotation is higher when only one type of analysis, sequence- or structure-based, is used when making predictions. As methods continue to be optimized and used in parallel with other methods, the information obtained though the genome projects can become more useful and complete. With the help of these breakthrough computational methods listed above and others to come in the future, the challenges of assigning functions to proteins can begin to be resolved. Even with the number of methods available today to predict the function of proteins, it is clear that the field of protein function prediction will continue to grow, especially as the quality and quantity of data continue to increase. While these computational methods are being optimized, biochemical studies can be used to validate the predictions made. Such experimental verification is a major current need in the field. In the future, as computational methods improve and are subjected to experimental verification, biochemical studies can be more focused and less time consuming. Future automation of the computational methods will enable fast, high-throughput functional annotation of these proteins and thus add significant value to the vast, growing store of genomics data.
